# The ABCs of mental health at the university: a multi-level intervention design for promoting mental well-being

**DOI:** 10.3389/fpubh.2024.1382393

**Published:** 2024-10-09

**Authors:** Line Nielsen, Elena Bermejo-Martins, Malene Kubstrup Nelausen, Cecilie Schacht Madsen, Elena Riva, Vibeke Jenny Koushede, Charlotte Bjerre Meilstrup

**Affiliations:** ^1^Department of Psychology, University of Copenhagen, Copenhagen, Denmark; ^2^Institute for Advanced Teaching and Learning, University of Warwick, Coventry, United Kingdom

**Keywords:** mental health promotion, mental well being, whole university approach, students, higher education, complex intervention

## Abstract

**Background:**

There is an escalating concern for the mental health of university students being recognized as a high-risk group for psychological distress. Despite research emphasizing the need to integrate mental well-being into higher education, existing interventions primarily focus on challenges and support services, leaving a gap in practical insights for promoting mental well-being at the university as a whole.

**Objectives:**

This paper aims to cover the theoretical and methodological foundations for the design and development of a complex multi-level intervention called the ABCs of mental health at the university (ABC-uni).

**Methods:**

Following the MRC framework for complex interventions, the design and development of a novel complex intervention is described (Phase I). Using the socio-ecological model and incorporating principles from health promotion charters, capacity building, organizational change models, and the principles of the ABCs of mental health a program theory for mental health promotion at the university is proposed. Following this theoretical foundation a logic model outlines the ABC-uni intervention components at structural, community, and individual levels. The components include staff training, campaign materials, a photovoice project, integration of mental well-being literacy into introductory programs, a credit-bearing course, and an online module. Preferably, most of these components are developed and carried out collaboratively with students at the university. As proposed in the logic model expected outcomes encompass heightened awareness, supportive environments, commitment, and accountability, aiming to enhance mental well-being across the entire university. The design and development of the intervention components occur at a Danish public university.

**Discussion:**

This section addresses the strengths and limitations of the design of the ABC-uni intervention. Future research will cover the feasibility phase of each components of the ABC-uni intervention (phase II). The conceptual framework and program theory outlining mental health promotion at the university, along with the detailed description of the intervention components, provides valuable insights for fostering mental well-being in the university community.

## 1 Introduction

### 1.1 University students’ mental health and well-being

Young peoples’ mental health and well-being have been a public health issue of increasing concern in recent years with a growing body of empirical research showing that university students are a ‘very high-risk population’ for psychological distress and mental disorders (1). When young people enter university, they are introduced to new demands and stressors that can profoundly affect their mental health and well-being (2, 3). To date, much of the research on university student well-being has focused on investigating the prevalence of psychological distress and on the use of support services for students experiencing mental health problems (1).

Mental health is more than the absence of mental disorders or disabilities, and can be seen as a continuum throughout life, encompassing positive aspects and our ability to connect, function, cope and thrive (4). In a salutogenic perspective focus is on positive aspects of mental health, also labeled mental well-being, and on protective factors for mental well-being, adding value beyond what is achieved from a pure risk reduction perspective (5, 6). This is outlined in the theory of salutogenesis emphasizing the concept of sense of coherence as an orientation toward life as being understandable, manageable and meaningful (6, 7).

Individual, family, community and structural factors may protect or undermine individuals’ mental health and change their position on the mental health continuum. As factors determining mental health and well-being are multisectoral, multi-level interventions to promote and protect mental health and well-being should also be delivered across multiple sectors and hence also in the higher educational sector (4). The socio-ecological model of mental health and well-being identifies several levels of importance when looking at protective factors: individual (e.g., mental well-being literacy, emotional resilience), community (e.g., sense of belonging, social support), and structural (e.g., supportive environment, social, economic, and cultural conditions) (8–10). Several studies have shown a correlation between high levels of mental well-being among university students and a spectrum of positive outcomes, including improved learning, reduced dropout rates, and elevated levels of life satisfaction (11–13).

Establishing supportive environments is recognized as crucial for promoting mental health and well-being, as individuals are significantly influenced by the structures surrounding them (14). This is also the case for students in higher education (15). Given that a substantial proportion of students will experience mental health difficulties during their time at university, it is important to consider how universities can ensure to provide supportive and ‘mental health-promoting’ environments (16, 17). Therefore, more research is needed on how to promote mental well-being in the university context.

### 1.2 Promoting mental well-being in the university context

Higher education holds a unique role in society with potential for providing transformative education, engaging student voices, and advocating for the well-being of individuals and communities. Positioned at the forefront of the knowledge society, universities have the potential to generate and implement knowledge to enhance public mental health both within and outside the university. Indeed, this appears highly pertinent, considering the association between mental well-being and learning (11). Poor mental health and low well-being can impact academic achievement (18), and low levels of resilience can limit a student’s learning capacity and engagement with consequences for continuation and attainment (19). At a more philosophical level, higher education is also about cultivating “a complete, integrated person” emphasizing the importance of incorporating a focus on mental well-being and not solely concentrating on academic aspects (20).

Embracing the principles of Health Promoting Universities, it is proposed to integrate health into everyday operations, aligning with Mental Health Promotion (MHP) strategies (21, 22). To do this in a university setting, the five actions for health promotion proposed by the Ottawa Charter (23) can be applied. As described in the Okanagan International Charter for Health Promoting Universities and Colleges (22), the following actions are specifically tailored to the university setting:

1. Embed health in all campus policies: ensure policies focus on health, well-being, and sustainability across individuals, campus, community, and environment.
2. Create supportive campus environments: enhance the campus as a living laboratory for studying and supporting health, well-being, sustainability, and resilience.
3. Generate community action and a culture of well-being: foster empowered, connected, and resilient campus communities that prioritize care, compassion, collaboration, and community action.
4. Support personal development: build resilience, competence, and life skills in students, staff, and faculty to help them thrive as engaged citizens.
5. Create or re-orient campus services: design services to support equitable access, enhance health and well-being, and promote a supportive organizational culture.

### 1.3 A whole university approach

This aligns with the concept of a ‘whole university approach’ emphasizing the intricate interrelation of students’ living conditions, learning environments, access to support, and sense of community in influencing mental well-being (24). This approach underscores that mental health support should extend beyond isolated services provided by specialist teams and thus should be integrated into all aspects of university.

In schools, contemporary frameworks for MHP frequently involve whole-school approaches, such as the World Health Organization’s ‘whole school approach to mental health promotion’. This commonly used approach is based on three interrelated pillars: curriculum, teaching and learning; the school ethos and environment; and family and community partnerships, which presents a clear and manageable framework for schools to engage with (25), and which bears relevance to the higher education sector. It is notable that a crucial element of this approach is ‘curriculum, teaching and learning’. Thus, it is unsurprising that embedding mental well-being in the learning experience of higher education students has been recognized as an opportunity for a holistic, student-centered approach to mental well-being and that it is considered as an important element of the whole university approach (21). Indeed, the curriculum, identified as the sole assured point of contact between students and the university, presents a valuable avenue for implementing preventive measures to enhance student mental health and well-being (26). This involves adopting inclusive pedagogical approaches (13), embedding well-being interventions into their curriculum in the recognition that teaching, pedagogy, and assessment practices can support higher education student well-being (1) and including (or ‘infusing’) discipline-relevant mental health-related content into the curriculum (27), increasing student mental well-being literacy understood as vocabulary, knowledge and language skills that may be intentionally used to maintain or improve mental well-being of oneself or others (28). Strong peer connection, teacher-student interaction (13), and communication are also pivotal to well-being and learning (29).

Moreover, working in partnership with students is increasingly recognized as an important aspect of a whole university approach to mental well-being (30), and participatory approaches are increasingly prioritized in the sector. In the UK, the University Mental Health Charter (21) requires universities to work in partnership with students throughout the different dimensions of the framework, and examples of staff-student partnerships are becoming increasingly common. Lister et al. (31) describe co-creation projects in two universities to create resources and initiatives to support student well-being. In the same line, the classroom (be it online or face to face) and teaching practices contribute to experiences of mental health and well-being (13, 32). Importantly, all the aspects of the student learning experience, from the classroom culture to the physical learning spaces, from the course design and curriculum content to the assessment and feedback strategies, from the group work and teacher-student dynamics to the available online support can impact on student mental health and well-being in a positive or negative manner, and can act as enablers or barriers to student mental well-being (33). Moreover, it has been observed how student-centered learning experiences that hinge on active learning and student participation sustain student well-being (13, 31).

In the pursuit of the shared objective to enhance mental well-being and apply a whole university approach including individual, community, and structural levels, we propose to use a clear framework for MHP.

### 1.4 The ABCs of mental health: a research-based framework for mental health promotion

The ABCs of mental health (ABC) provides a research-based framework and principles for MHP. In Denmark the work with implementing the framework is organized as an interdisciplinary and multisectoral partnership called the ABC-partnership. The ABCs is inspired by Act-Belong-Commit, a universal MHP initiative developed at Curtin University, Western Australia (34). The ABC-partnership aims to (1) increase knowledge and understanding of what individuals can do to strengthen their own and others’ mental health and well-being and (2) create and support the best conditions for mental health and well-being for the entire population across life circumstances and conditions. The approach involves utilizing research-based information, fostering capacity building, sharing knowledge, and promoting collaboration across various disciplines and sectors (35, 36).

The ABCs is theoretically rooted in a salutogenic perspective inspired by the work of health research pioneer Aaron Antonovsky (1923–1994) and his resource-and possibility-oriented understanding of health (37). The ABC-framework can be understood as a principle-based approach (38, 39). This indicates that the ABCs is not a standardized, manual-based intervention that follows specific procedures and is implemented uniformly across all settings. In a principle-based approach overarching principles guide the work, in this instance, MHP and can inspire innovation at the local level and provide guidance for adapting and implementing the framework (40). The five guiding principles in the ABCs are as follows:

1. Take a universal approach: mental health concerns everyone no matter their mental health status, socioeconomic background, age etc.
2. Use the ABC messages explicitly: (A) Do something, (B) Do something with someone and (C) Do something meaningful.
3. Adapt a salutogenic understanding of mental health and well-being: focus on protective and mental health promoting factors, resources, and settings.
4. Promote collaboration across sectors: mental health is something we create together.
5. Adapt initiatives locally: apply the framework to new or existing interventions.

A process evaluation among ABC-partners in Denmark highlighted that the framework provided relevant knowledge on MHP to stakeholders and fostered intersectoral and interprofessional collaborations by providing a common language for MHP (35). However, the evaluation also pointed out that its bottom-up approach requires time and resources, as well as a continuous deliberate balance between local adaptability and concrete guidance when engaging implementers. A study evaluating the impact on approximately 3,000 randomly chosen individuals in Denmark revealed that those familiar with ABC gained new insights, engaged in self-reflection, and became more open to discussing mental health with others (36). Additionally, the awareness of ABC promoted mentally healthy behaviors. The analyses included both individuals with and without mental health issues, showing a consistent trend, with slightly higher figures observed among those experiencing mental health problems. Finally, a recent report from the OECD, included ABC as a case study representing an integrated approach to MHP (41).

Despite the positive experiences reported, there is a notable gap in understanding how ABC can be adapted and implemented within a university context.

### 1.5 Strategies and mechanisms for capacity building for mental health promotion at the university

To implement the ABC-principles there is a need for capacity building at various levels at the university. In the health promotion literature capacity building has been defined as “*an approach to the development of sustainable skills, organizational structures, resources and commitment to health improvement in health and other sectors, to prolong and multiply health gains many times over”* (42).

In order to implement capacity building for MHP across various levels (e.g., students, campus, university staff and faculty members), five strategies have been identified as critical: leadership, infrastructure and organizational change, workforce development, partnerships and resource allocation (43, 44). To operationalize these strategies, the theoretical work by Hernantes et al. (45) about intersectoral action and building capacity for health promotion can be adapted to the university context. We therefore propose that the ABC-principles could stimulate the promotion of mental well-being at university through five mechanisms: a shared vision, using a common language, creating a supportive environment, and adopting participatory practices.

These mechanisms will enhance the engagement of the university community in promoting mental well-being and strengthen the sense of belonging to a culture of well-being among all members of the university context. Belongingness is the main crucial precursor of commitment (46), and when experienced collectively among students as well as staff, could help to ensure the sense of responsibility and accountability (45). As a result, we hypothesize that the above-mentioned mechanisms will lead to increasing awareness, knowledge, skills and resources among students, staff, and the broader university context. At the same time, they will increase the commitment and accountability of various stakeholders and university decision makers (e.g., university board, heads of departments and deans) by helping them to understand that mental well-being issues is a shared responsibility which cannot solely be solved by focusing on treatment and prevention, but also needs to focus on promotion and take actions accordingly.

### 1.6 Developing a comprehensive and multi-level intervention to promote mental well-being

Despite recommendations, positive experiences, and knowledge on MHP, there is a gap in understanding the design and implementation of a whole university approach to students’ mental well-being: What should the design encompass at which levels, how should it be shaped, and how should it be delivered and implemented? In other words, there is a lack of practical and tangible insights into promoting mental well-being among university students. Recognizing this gap there is a need to develop comprehensive and multi-level interventions to promote mental well-being in the university setting, integrating the perspectives and frameworks mentioned earlier.

### 1.7 Aim

The aim of this paper is to describe the design and development of a multi-level intervention aimed to promote mental well-being at the university following the MRC framework for complex interventions. The focus is on Phase I covering a comprehensive description of the conceptual framework for mental health promotion at the university as the program theory followed by a logic model to be tested in a future feasibility study (Phase II).

## 2 Methods and materials

### 2.1 Study design

The MRC-/NIHR-funded INDEX study has developed a comprehensive framework and guidance on complex intervention development and evaluation (47). Their proposed methodological framework entails four main phases of intervention research: development or identification of the intervention, feasibility, evaluation and implementation. Each phase is connected to a set of six core elements considering context, developing and refining program theory, engaging stakeholders, identifying key uncertainties, refining the intervention and economic considerations. These elements are revisited continually throughout the research process and especially before moving between phases (e.g., between feasibility assessment and evaluation) (48).

This study encompasses Phase 1 that involves the development and theoretical underpinning of the intervention. This phase focuses on understanding the problem, identifying potential solutions, and creating the sound program theory and logic model for the intervention to be tested in the following feasibility study (Phase II). It’s important to note that program theory and logic models are not static. They should be tested and refined throughout the development process using primary and secondary data collection and stakeholder input. Indeed, they are advocated for use in process evaluations alongside outcome evaluations in the recent MRC Guidance on process evaluation (48).

### 2.2 Study population, setting and participant engagement

The intervention adopts a multi-level and whole university approach, and study participants include staff and students at the FSS at the University of Copenhagen. The student body consists of roughly 6,600 students enrolled across 7 bachelor’s and 10 master’s programs in anthropology, psychology, social science, sociology, political science, and economics. There are about 630 employees (excluding part-time scientific personnel) (49).

The proposed intervention is called the ABCs of mental health at the university (ABC-uni). The study is conducted at the Faculty of Social Sciences (FSS) at the University of Copenhagen (UCPH) in Denmark. The higher education system in Denmark is characterized by a student-centric approach, offering a three-tier-structure comprising bachelor’s, master’s, and doctoral programs. In Danish universities there is a widespread adoption of problem-based learning with the intent to foster critical thinking and teamwork. While tuition fees apply to non-EU/EEA students, Danish and EU/EEA students benefit from free education (50). Participation in most ABC-uni components, such as online and physical courses, is self-selected and voluntary, while other components, like the photovoice exhibition or teacher training, have a more universal nature. A more detailed description of participants and engagement strategies will follow in the description of the intervention components.

### 2.3 The program theory of the ABC-uni

As part of Phase I of a complex intervention development, the creation of a robust and clear program theory is critical (48). The goal is to describe how an intervention is expected to lead to a set of specified outcomes and under what conditions. It articulates (1) the key components of the intervention and how they interact; (2) the mechanisms of the intervention; (3) the features of the context that are expected to influence those mechanisms; and (4) how those mechanisms may influence the context and lead to achieve the desired outcomes.

**Figure 1:**
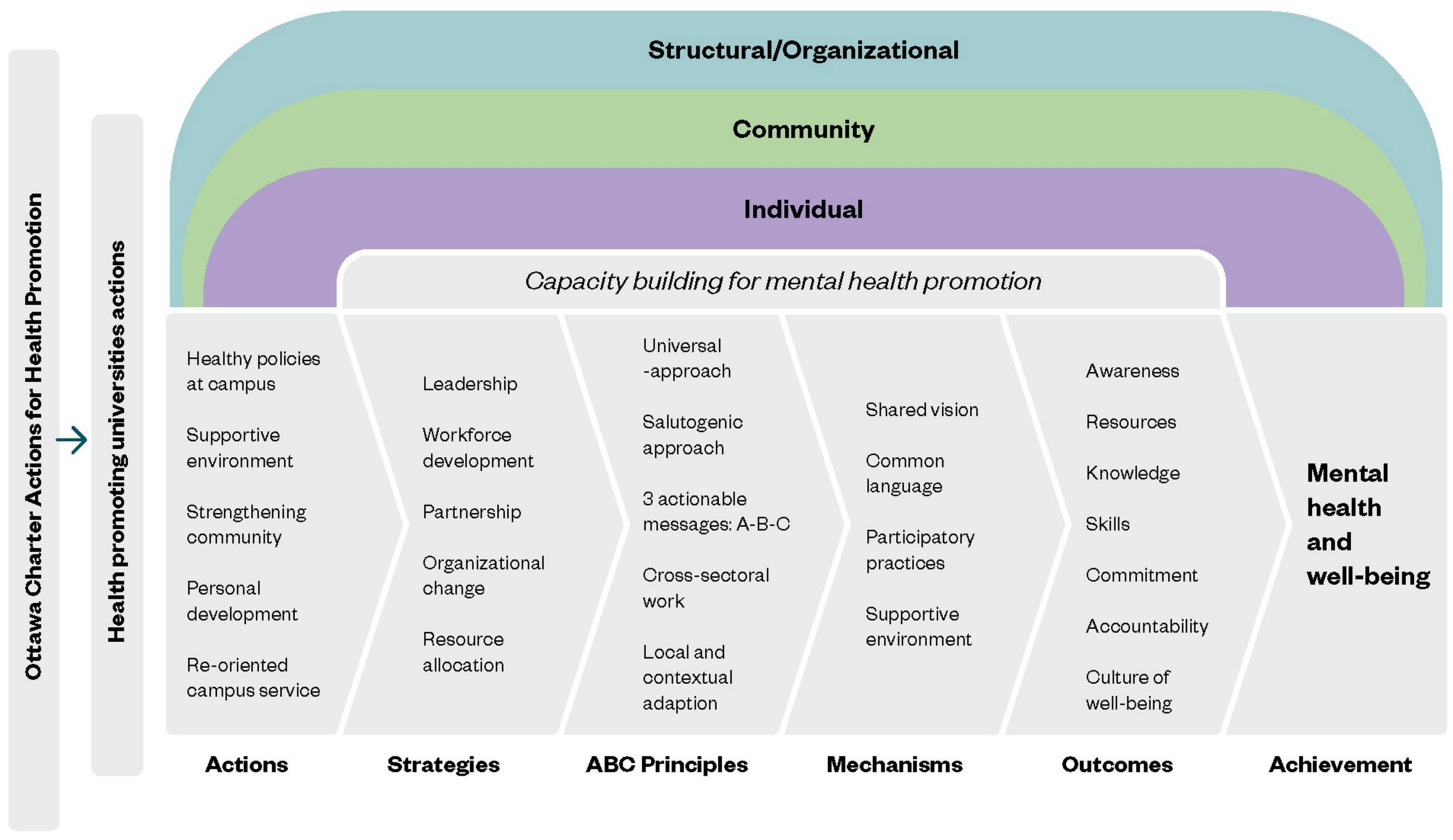
The program theory of ABC-uni showing the theoretical foundation and the process of change to be implemented.

Figure 1 articulates the different theories and models that have been presented in the introduction as underlying the strategy for building capacity for MHP in the university context. The proposed conceptual framework for mental health promotion at the university highlights the proposed five actions from the Ottawa Charter for Health Promotion into the university context by Okanagan International Charter for Health Promoting Universities and College: healthy policies at campus, supportive environment, strengthening community, personal development, and re-orienting campus services. These actions lead to five strategies necessary for a capacity building process for promoting mental well-being: leadership; workforce development; partnerships; organizational change; and resource allocation. First of all, it is critical that leadership for the advancement of MHP is apparent at the level of the board and management team as it enables strategic vision and planning. Second, the development and sustainability of MHP is dependent on having a skilled and informed workforce (in the university context, it extends to also include the students). Therefore, workforce capacity development can range from increasing mental well-being awareness and literacy to training and skills development needed to support and implement specific initiatives (51). Third, the partnership component requires developing and implementing multiple strategies to engage and facilitate the participation of diverse sectors (in the university context, of, e.g., various disciplines, departments, student-led organizations and initiatives, and subgroups) (8). And lastly, creating organizational change with a shared understanding of the organization’s well-being goals and strategies, including allocated resources, are required, in order to make MHP work effectively and sustainably.

The ABC-framework and messages (Act, Belong, Commit) provides key pragmatic principles: using a universal and salutogenic approach, spreading an actionable message through the implementation of activities and materials, adopting a cross-sectoral action (multi-disciplinary) and local adaptation (within the university context). According to Kaluzny’s (52) Organizational Change Model (adapted to the university context) this process of change may involve four sequential stages: awareness, adoption, implementation, and institutionalization. The ABC-principles are the core motor in articulating the strategies for capacity building into key mechanisms of change by developing a shared vision, adopting a common language, engaging in participatory practices and lastly, creating a supportive environment with a strong sense of belonging.

This process should lead to the main expected outcomes at three different levels:

1. At the structural level (leaders, faculty members, administrative staff, and stakeholders at FSS): increasing their capacity for MHP.
2. At the community level (members of the campus at FSS): enhancing available resources and creating a culture of well-being.
3. At the individual level (students and tutors (peer-mentors) at FSS): increasing mental well-being awareness, knowledge, skills, and resources.

As a result of this process, the end goal is to increase mental well-being at the university as a whole.

### 2.4 The logic model of the ABC-uni intervention

**Figure 2:**
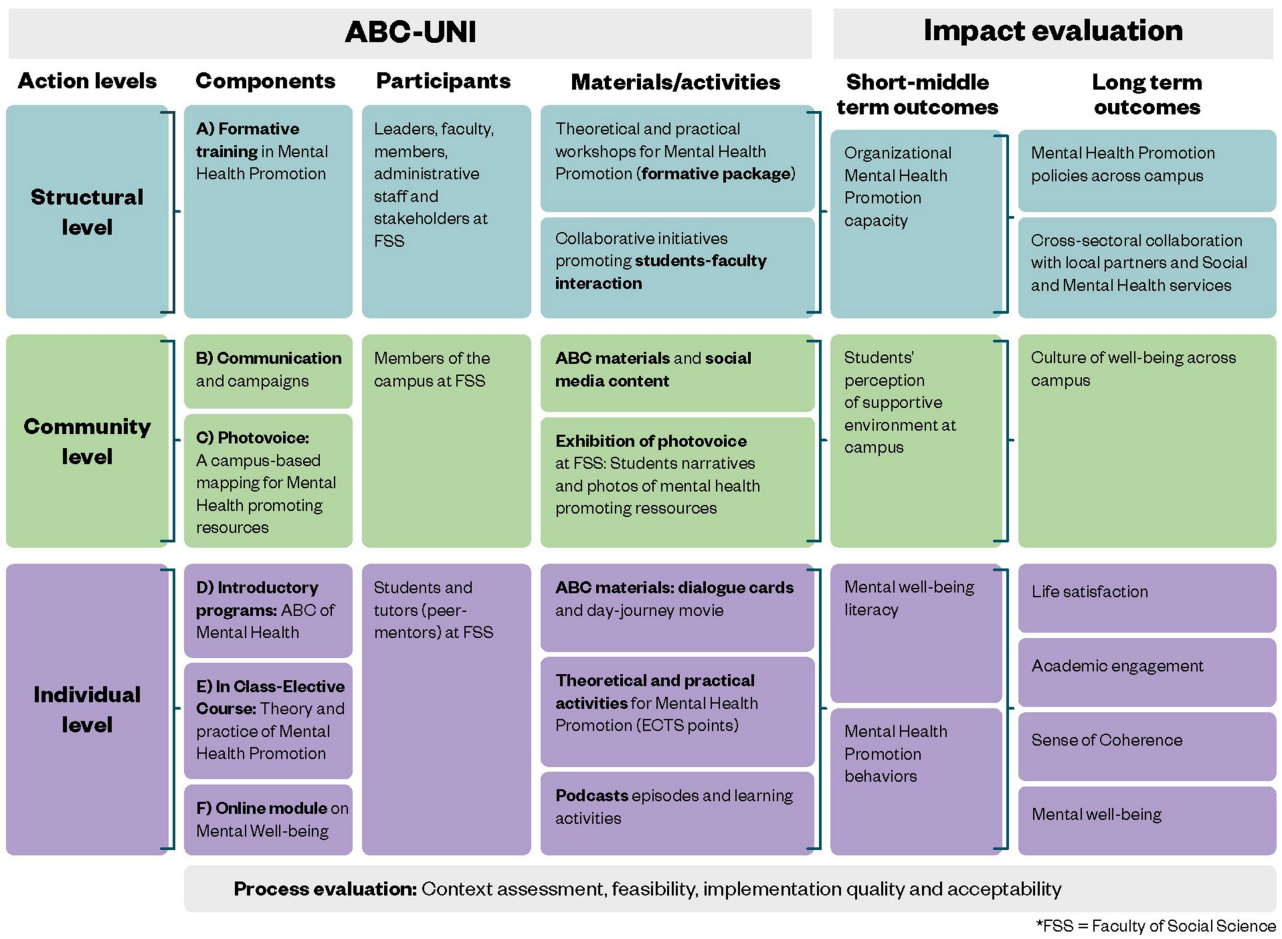
The logic model of the ABC-uni showing the action plan for each intervention component and the expected outcomes.

The logic model has proven to be a successful tool for program planning as well as implementation and performance management in numerous fields including public health (53). It is defined as a graphical and textual representation of how an intervention or program is intended to work. It links outcomes with processes and the intervention’s theoretical assumptions. Building on our proposed program theory for mental health promotion at the university, the aim of the logic model in Figure 2 is to make explicit the theory of action underlying the ABC-uni intervention and provide a graphical common approach to integrate the planning, implementation, and future evaluation of each component of the intervention.

Figure 2 shows the relationships between the different action levels (structural, community and individual) at which the intervention is targeted, its actionable components connected to specific participants and the activities involved. The activity-design is a full expression of the mechanisms of change and active ingredients of the intervention by: ensuring a shared vision on promoting mental well-being; using a common language with the ABC-principles; adopting participatory practices during the design and implementation of activities, and creating a supportive learning environment.

All the above elements relate to the main proximal (short and middle-term) and distal (long-term) expected outcomes from each component in the intervention. The outcomes across the three actions levels of the intervention reflect the specific way to ensure the achievement of the general outcomes described in the conceptual framework:

- Structural level: increased awareness, knowledge, skills, resources, commitment and accountability by assessing the organization capacity for MHP and promoting actions for mental well-being policies in the university agenda.
- Community level: improvement of the study environment and culture of well-being by assessing perceived supportive environment and identifying mental well-being resources at campus.
- Individual level: increased awareness and knowledge by measuring mental well-being literacy; skills and resources by assessing mental health promoting behaviors, sense of coherence, life satisfaction and commitment by measuring academic engagement.

Therefore, this logic model reflects a series of “if then” relationships that, if implemented as intended, expectedly will lead to the desired outcomes. However, it is essential to comprehend the logic model in the context of resources and collaboration acting as crucial inputs for the success of starting up the intervention. Key elements encompass university board support, internal funding, project-specific budget allocation, time, personnel, resources, and the establishment of a cohesive research team. The collaboration covers internal cross-collaboration at the University of Copenhagen: student volunteers, student assistants, student organizations, communication office, data management, educational advisors, student counseling service and Center for Online and Blended Learning; and external cross-collaboration: Institute for Advanced Teaching and Learning at University of Warwick, the ABCs of mental health partnership in Denmark and Studenterrådgivningen (national student counseling service in Denmark).

### 2.5 Description of the components in the ABC-uni intervention

The following sections provide a detailed description of the design of the various components of the designed complex intervention including background, objectives, participants and materials and activities. Whereas some components are already developed and ready to be tested in the feasibility study (Phase II), the development of others are still in progress. This accounts for the differences in the level of detail provided.

The intervention components have been be developed by the research group behind ABC-uni in collaboration with several cross-collaborators at the FSS including student volunteers, student assistants, student organizations, tutors, communication officers, educational advisors, and student counselors.

#### 2.5.1 Formative training in mental health promotion for staff

##### 2.5.1.1 Background

According to the whole university approach, both academic and administrative staff, alongside counseling personnel, play crucial roles in the comprehensive strategy. This perspective recognizes the collective importance of all staff members in contributing to the well-being of the university community. A study among students at a campus-based metropolitan university in Australia focused on how universities can enhance student mental well-being with the majority of student recommendations focusing on the importance of academic teachers and teaching practices (1). A qualitative study involved interviews with professors recognized for their teaching excellence and focused on mattering in the teacher-student relation as a way to strengthen the learning, development and well-being of students (54). Educational advisors and student counselors also play a pivotal role in the daily lives of students, serving as essential bridges to impart knowledge, competencies, and support (55). Through their regular interactions with students, these professionals can contribute to the creation of a safe and inclusive campus environment that extends beyond academic guidance, encompassing the vital task of nurturing a supportive atmosphere that can contribute to the mental well-being among students.

In the ABCs, the capacity building efforts aim to increase knowledge about MHP and improve relevant organizational structures, e.g., through the training of employees and volunteers, and creating local and national intersectoral and interprofessional collaborations. A process evaluation has shown that it can be particularly effective to engage non-health-oriented implementers by presenting MHP and the ABC-framework as means of supporting them in conducting their core-tasks, which might not be explicitly linked to mental health and well-being, which is the case for most of the faculty staff (35).

##### 2.5.1.2 Objectives

The formative training of staff aims to enhance knowledge and competencies in MHP using the ABC-framework, fostering the capacity to create supportive environments for mental health and well-being within the university. Objectives include elevating staff knowledge, skills, and resources to cultivate a shared language for MHP and to integrate a well-being focus in teaching and counseling practices.

##### 2.5.1.3 Participants

The formative training in MHP will target faculty and staff, including educational advisors and student counselors. Various engagement strategies will be employed based on the specific type of formal training. The participation rate will significantly hinge upon the availability of leadership endorsement and the level of interest demonstrated by faculty and staff members in participating. It is anticipated that approximately 30 faculty or staff members will participate in the action learning processes. Additionally, it is expected that around 25 adjunct lecturers each year will become familiarized with a focus on well-being at the university and mental health promotion, including exposure to the resources provided by ABC-uni, as part of their teacher training program. Should the online module be extended to faculty and staff members (see 2.5.6), it is projected that roughly 5–10% of faculty and staff will actively engage with the podcast episodes. This projection is informed by a recent deployment of an online module concentrating on cyber security to all faculty and staff members at the faculty, which exhibited a notably low participation rate (no reference available).

##### 2.5.1.4 Materials and activities

Action learning processes will be developed with academic and administrative staff. The advantage of an action learning program is that it can contribute to local development processes and serve as a valuable bridge between theory and practice. Positive experiences with ABCs action learning programs demonstrate their scalability, adaptable to varying resources and ambitions (35). ABC-uni will build upon initial experiences from an action learning program for educational advisors and student counselors at the FSS, which will be unfolded over three workshops and aimed to build knowledge and competencies to enhance MHP in the educational work at the university and in student counseling services.

The Teaching and Learning in Higher Education Program at the FSS is a one-year course designed to develop and upgrade university teachers’ pedagogical and didactic skills. A perspective on student well-being is included as a new theme in the training from 2023. This is an opportunity to create awareness of the ABC-uni and build participants’ knowledge and skills to promote mental well-being in the teaching and learning environment also building on the findings of mattering in teaching by Phycyl et al. (54).

The different types of formal training in MHP will, if possible, also include a perspective on student-staff-interaction to increase knowledge on how these interactions can contribute significantly to academic and personal development (1, 13). Inspired by the principles of the ABCs, the training will also include specific examples on how student-staff-interaction can be fostered. These illustrative examples are drawn from the Department of Psychology, where student assistants have engaged in diverse events and initiatives (56) e.g. conducting workshops with students and staff together to collaborate on the design of a more inclusive and diverse curriculum; guest lectures inviting both faculty members and students; using social media to present faculty staff to students and providing spaces for casual and interactive exchanges and feedback sessions where students can share their perspectives with staff.

Finally, it is being considered to encourage all staff members to engage in the online module intending to foster mental well-being literacy (see 2.5.6). Even though the module is developed for and in collaboration with students, the content of the themes and podcast episodes may still be relevant for staff in terms of building knowledge, skills and a common language.

#### 2.5.2 Communication and campaigns

##### 2.5.2.1 Background

Raising awareness, knowledge and skills to promote mental well-being can contribute to increasing mental well-being literacy (57). Experiences from the ABCs have shown that awareness of the campaign has prompted individuals to reflect on their mental health, engage in conversations about mental health with friends and family, and take action to enhance their own mental health (36).

##### 2.5.2.2 Objectives

The objectives of developing campaign materials are to create awareness and increase mental well-being literacy at the individual level and to foster a mental well-being culture at the community level.

##### 2.5.2.3 Participants

The target groups of the campaign materials are all students and staff at campus. According to the different types of materials developed, the target groups differ: Campaign films featured on campus info screens will be visible to everyone walking around campus whereas dialog cards and the day journey movie would most often be presented to students in student cafés, as part of working in study groups or through various events at campus.

Anticipating the scope of expected participants poses a considerable challenge. It is presumed that a majority of the students and faculty members at FSS will encounter communication and campaign materials at some point. Nevertheless, it is anticipated that only a minority, approximately 10%, will retain awareness or pursue further engagement with the initiatives of ABC-uni due to exposure to campaign or communication materials. This assumption is based on the findings from an impact evaluation of the ABCs of mental health campaign in Denmark (36).

##### 2.5.2.4 Materials and activities

Physical and digital materials: In the ABC-partnership campaign materials have been developed with different themes every year and spread through the ABC-partners’ different channels and platforms (SoMe, websites, public spaces etc.) to raise mental well-being literacy in the public. As part of the ABC-uni intervention, campaign materials are developed continuously and will be inspired by the campaign materials developed and used in the ABC-partnership. The materials will include a spectrum of resources, ranging from digital content optimized for social media platforms sharing the ABC-message, to items such as physical dialog cards.

Day journey movie: In the ABC-partnership, there is valuable experience in developing and utilizing a day journey movie to initiate reflections and conversations, sparking awareness and enhancing well-being literacy. In the ABC-uni, a day journey movie has been developed, inspired by the original movie but adapted to reflect university students’ everyday life (58). The movie was developed in close collaboration with five students at FSS who described a typical day in the life of a student. The students also took part in developing the script for the movie to make sure that their perspectives were included, secure an appropriate use of language etc. The narration in the movie is also done by a student who was actively involved in crafting the script for the movie.

The movie seeks to convey an appreciation for small actions, termed as micro-actions, directed toward oneself, others, and the community. These micro-actions encompass seemingly insignificant gestures, such as taking a break, rectifying a forgotten bicycle light for someone else, or offering a cup of coffee to a fellow student. The underlying concept is to demonstrate that various factors and actions possess the capacity to impact individual well-being and interpersonal dynamics. The movie shows that mental well-being is not only an individual matter but is also significantly influenced by the surrounding environment. In this regard, the movie places emphasis on the cultural and structural aspects within the academic setting, which are denoted as microstructures. By exploring these microstructures, the movie seeks to highlight their role in shaping the mental well-being of individuals, drawing attention to the broader context in which daily actions and interactions unfold. The day journey movie is preferably to be shown when facilitating a dialog among students on how they can initiate micro-actions on different levels, that may influence their own and others mental well-being.

Dialog cards: Physical dialog cards featuring 38 diverse questions centered around mental well-being and study life have been developed also with the aim of strengthening mental well-being literacy and foster conversations on mental well-being at campus. The dialog cards can either be used on their own or in combination with the day journey movie. The cards have been tested and customized according to feedback from students. The cards will be handed out to stakeholders at campus (e.g., heads of departments and student counselors) and be placed in different spots at campus (e.g., in the canteen and student cafés) to spark interest and awareness among students and staff. The cards have a QR code printed on them, enabling people to read more about the ABC-uni intervention. Additionally, it is the plan to create online versions of the cards and exploring the customization of new card sets especially focusing on working in study groups.

Communication through social media, info screens and physical materials: The universal campaign materials developed as part of the ABC-partnership aiming at spreading the ABC-message will also be used around campus at info screens, posters, featured in newsletters, the faculty intranet, and social media. This is part of raising awareness and conversations around mental well-being. Roll-ups with the ABC-messages and QR-codes have also been produced to be used as an eyecatcher at various events, e.g., the annual campus day.

#### 2.5.3 Photovoice project on mental health promoting resources at campus

##### 2.5.3.1 Background

The photovoice component, rooted in participatory action research (PAR) principles, empowers individuals to capture and convey their community’s perspectives through photography (59).

Studies find that the photovoice methodology is a powerful tool when working with different kinds of students as it empowers them to be more aware of their surroundings, amplify their voices in communication to university leadership, initiate change in the culture of the university, and represent strengths and challenges of the university community (60–63). Studies also show that the incorporation of visual images, can lead to a deeper understanding of the human experience than words alone and facilitate a safer space for reflections and discussions (64), which clarifies what is needed to promote mental health and well-being in everyday life of students at campus.

In this context, photovoice will be applied as a way to engage students to reflect on and document what they consider valuable resources for promoting mental well-being on campus, referred to as Mental Health Promoting Resources (MHPR). The resulting photographs will be showcased in a campus exhibition, providing a platform for students and staff to interact with the visuals and accompanying texts. This exhibition will serve a dual purpose of disseminating findings to faculty stakeholders, students, and staff, while also directly communicating these insights to faculty leaders and key personnel dedicated to enhancing student well-being through structural support and student engagement.

The initiative draws inspiration from the ABCs of mental health in two key dimensions:

1. Using the ABC-principles in the research design: working together with students as co-researchers, employing a salutogenic approach and using the ABC-messages to reflect upon the MHPR shown in the photographs taken by the students.
2. Using the ABC-messages in the exhibition to communicate to students, staff, and stakeholders, how to strengthen one’s own and others mental well-being on an individual-, community-, and structural level.

##### 2.5.3.2 Objectives

The aim of the photovoice component in the ABC-uni is to explore and understand students’ perceptions of MHPR crucial for enhancing mental well-being on campus. Recognizing the significance of comprehending these resources, the project seeks to bridge the gap in understanding how university environments influence student well-being. By focusing on students’ perspectives, the aim is to identify and highlight key factors contributing to mental well-being within the university context.

##### 2.5.3.3 Participants

The recruitment process seeks to engage students from all five departments at FSS to have different perspectives represented from each institute. The minimum number of participants recruited to carry out the photovoice project is set at 10 students, as the wish is to have diverse perspectives represented and enough students to participants to engage in focus group discussions. The maximum number of participants is 15 due to the available resources allocated to this component. Students that will participate in the photovoice project will be regarded as co-researchers, collaboratively contributing alongside university researchers in an evolving and relational approach to mutual learning. This approach is built upon the researchers willingly relinquishing complete control, as discussed by Phillips et al. (65). In this study, students will be engaged as active co-researchers when using photography to capture their perspectives on MHPR within the university setting and when analyzing the findings.

##### 2.5.3.4 Materials and activities

The project design entails a series of three workshops, aiming to cultivate discussions and impart knowledge about MHPR. These sessions will guide students in utilizing photography to encapsulate MHPR on campus and select photographs for an exhibition. Thematic analysis of the captured photographs and their accompanying texts will facilitate the identification of key factors pivotal to understanding the necessary components of MHPR crucial for establishing a mental health-promoting environment (66).

#### 2.5.4 Integrating a focus on mental well-being in introductory programs

##### 2.5.4.1 Background

The transition into university can cause a significant source of stress during the initial phase of university life (3). This period places intense strain on students’ well-being as they grapple with the challenges of adjusting to the new academic and social environment. This transition is an important window of opportunity to reach individual students through introductory programs and have a particular focus at community level on a culture of well-being at campus. A qualitative study from the UK on student mental health and transitions into, through and out of university, showed that students highlighted a need for universities to equip them with coping skills especially during the transition into university (67).

##### 2.5.4.2 Objectives

At the individual level, this component aims to enhance mental well-being literacy among students, with a specific emphasis on student life within the academic setting. At the community level, the objective is to establish a supportive and inclusive study environment from the outset of the academic journey.

##### 2.5.4.3 Participants

This component primarily targets students who are new to the university. Each year, approximately 1,000 new students enroll in the study programs offered at FSS. They are provided with the opportunity to participate in the digital and physical onboarding program, which also includes materials related to ABC-uni. Participants also include those transitioning from the BA program to the MA program, as well as students entering the university during their education from other institutions. Although it is challenging to anticipate what to expect, it is assumed that more than half of the new students will encounter ABC-uni materials with approximately 20% of them choosing to further engage with the approach, e.g., by enrolling in the online module (see 2.5.6).

##### 2.5.4.4 Materials

The FSS offers all new students a digital onboarding program that provides an introduction and essential tools for their entry into the university. This initiative encompasses online resources to help students understand digital platforms, access course information, and explore academic opportunities. Additionally, a section focusing on student life and well-being features the ABC-uni, and students are encouraged to participate in the online module promoting mental well-being literacy.

Tutors (peer-mentors) are more experienced students who are responsible for conducting introductory programs, guiding, and assisting new students as they navigate their initial experiences at the UCPH. At the faculty, tutors undergo training to conduct physical in-class introductory programs for new students. Tutors at FSS will also receive training in MHP and be provided with materials, such as dialog cards and the day journey movie mentioned earlier, for use in workshops during introductory programs. The intention is to highlight the importance of a culture of well-being, fostering conversations on this topic and facilitating a shared language on mental well-being.

#### 2.5.5 Elective credit-bearing course on mental health promotion in theory and practice

##### 2.5.5.1 Background

The curriculum, identified as the sole assured point of contact between students and the university, presents a valuable avenue for implementing preventive measures to enhance student mental health and well-being. Aligned with the whole university approach, interventions embedded in the curriculum and pedagogy can offer a universal approach (21). However, there is still a lack of research in this field. A systematic review of curriculum-embedded interventions targeting student mental health and well-being examined 46 longitudinal pre−/post-studies from June 2015 to May 2020, and found that most interventions did not significantly impact stress or anxiety (29). The authors emphasize the need for increased funding to enhance study power through collaborative efforts and call for improved reporting quality to facilitate robust meta-analyses and more conclusive findings.

An important aspect of embedding mental health and well-being in the curriculum and in the learning experiences of students is to implement curricular but also extracurricular interventions that aim at increasing students’ mental health and well-being literacy. Supporting mental well-being literacy constitutes an important aspect of MHP. In Ireland, Hill et al. comment that “*A key element of a whole system approach is education and training in mental health literacy for staff and students, to help identify signs of distress and direct students to appropriate resources*” (68). Interventions to increase mental health and/or well-being literacy can be found in several different European contexts. For example, a study using a video intervention found non-significant positive trends toward increased mental health literacy with 101 university students in France (69). The University of Warwick has recently developed online and in-person courses aimed at increasing the mental health and well-being literacy of students (70). In the module, students explore the concept of well-being from different disciplinary viewpoints, ranging from scientific, economic, psychological, and philosophical perspectives to create a holistic understanding of this complex concept. The students also engage with activities and strategies that can improve their well-being, e.g., exercise, art, and mindfulness.

##### 2.5.5.2 Objectives

The objective of this component in the ABC-uni is to embed mental well-being and MHP in the curriculum, and to provide students with a fundamental understanding of MHP and equip them with analytical and theoretically grounded skills. The course aims to enhance students’ knowledge, competencies, and capabilities to promote mental well-being, serving as both an immediate resource and a supplementary skillset to their broader academic expertise developed throughout their studies. Upon course completion, students will gain comprehensive knowledge of mental well-being and they will learn theoretical and methodological approaches to define and measure mental well-being and explain influencing factors at individual, community, and structural levels. Students will cultivate competencies in analyzing cases, proposing solutions, and engaging in interdisciplinary discussions on MHP. The active learning process is intended to also contribute to raise mental well-being literacy among the students (71).

##### 2.5.5.3 Participants

The elective course is anchored at the Department of Psychology and offered across the FSS at UCPH. The aim is to engage students from different disciplines in the topic of mental health promotion, emphasizing the interdisciplinary nature of the student body. It is anticipated that the course will be offered annually with a maximum capacity of 30 students for each iteration. The recruitment of students follows the standard procedures for courses offered at UCPH. This course is included in the course catalog accessible to all enrolled students at FSS. Additionally, the elective course will be featured on the ABC-uni webpage and communicated to students upon completion of the online module (see 2.5.6) as an opportunity to delve deeper into the scholarly aspects of mental health promotion.

##### 2.5.5.4 Materials and activities

This interdisciplinary elective course explores MHP from various professional perspectives. Students are acquainted with different theoretical frameworks and perspectives on mental health, including social-psychological, socio-economic, public health, and educational perspectives. Various empirical studies utilizing qualitative and quantitative methods will also be presented. The course adopts a problem-and project-oriented approach inspired by action research, with a continuous interplay between theory and practice. Each teaching session will typically combine lectures and practical exercises, actively integrated into students’ projects. Throughout the course, students will engage in student-led presentations, group discussions, exercises, and reflective in-between-classroom activities. This structure allows students to apply their knowledge, competencies, and skills in practice. In interdisciplinary project groups, students will work on a self-selected case, allocating time for this work between teaching sessions.

#### 2.5.6 Online module promoting mental well-being literacy for all students

##### 2.5.6.1 Background

Web-based interventions have become more widespread within recent years (72, 73). There are several advantages, e.g., they can reach a wider and larger group of young people, reduce stigma, be approached flexibly and anonymously and be cost-effective. A meta-analysis on web-based interventions for students on mental health in higher education revealed effectiveness in reducing depression, anxiety, and stress symptoms compared to control groups (74). The interventions also improved interpersonal relationships and perceived social support. However, further research is necessary to confirm the robustness of these effects across diverse target groups and over time.

Online courses teaching well-being seem to have potential to promote mental well-being as demonstrated in a UK-based longitudinal study involving over 1,180 participants who took part in a psychoeducational happiness course at the University of Bristol (75). A large study of online courses compared well-being levels among six cohorts of adult students before and after they completed either a science of well-being course or a control course on introduction to psychology (76). The findings indicated that online courses teaching evidence-based well-being approaches have the potential to positively impact mental health and well-being of the students.

The online module in the ABC-uni intervention is strongly inspired by the online module ‘Understanding well-being’ developed and delivered by University of Warwick. In the ABC-uni online module students will gain insight into interdisciplinary and bio-psycho-social aspects of mental well-being as well as knowledge about MHP at the individual, community, and structural levels.

##### 2.5.6.2 Objectives

The learning objectives are to enhance students’ mental well-being literacy (knowledge, reflection, and action). By completing the online module, the student will gain knowledge about mental well-being and how it can be promoted, be encouraged to reflect on the concepts of mental well-being and MHP, acquire an understanding of the importance of the surrounding context for mental well-being, be presented to tools that aim at strengthening mental well-being (both at the university/campus and in their private life), and acquire skills to strengthen own and others’ mental well-being. A pivotal aspect of the online module is to inspire and motivate students to engage in activities and exercises that can impact their own and others’ mental well-being using the ABC-framework as an inspiration.

##### 2.5.6.3 Participants

The module is designed for students and is accessible through the educational platform Absalon, which is available to every student at UCPH. Students are recruited by creating awareness of the online module via the intranet, SoMe, posters at campus etc. Once students have signed up for the online module, they receive information on the research and evaluation and have the possibility to consent to participate. Participation in the online module as well as the evaluation of the online module is completely voluntary. The online module will not give ECTS points, but students who complete all podcasts and engage in the learning activities, can claim a course certificate.

All students at FSS potentially have the opportunity to enroll in and complete the module. Drawing from experiences at the University of Warwick, it is anticipated that around 10% of the students within the faculty will register for the module (approximately 650 students) with an estimated 20% of these students participating in the subsequent research study of reach and impact of the online module. The study will collect empirical data in form of a survey before and after students’ engagement with the online module and group interviews.

##### 2.5.6.4 Materials and activities

The online module covers 11 themes about mental well-being. Each theme includes a podcast episode with an expert interview, associated learning activity (quiz, reflection assignments and mini experiments), and suggested readings etc. The online module was designed, developed, and produced in collaboration and co-creation between our research group based at the Department of Psychology, students and The Centre for Online and Blended Learning (COBL) at UCPH. Throughout the co-creation process with students, a workshop was conducted during the design phase, engaging two students, the research group, and two COBL representatives. One student from the workshop became a podcast host together with one of the staff of the research group. Student perspectives were integrated into all podcast episodes through individual interviews with seven students from various UCPH study programs. The student host conducted these interviews, fostering openness and honesty in the student voices due to the peer-to-peer interaction. The student voices from these interviews acted as a reflective mirror, bridging the gap between theory and practice. The podcasts, enriched by students’ lived experiences, served as a foundation for conversations, offering listeners insights that translated expert knowledge into practical understanding from the students’ perspectives.

Benefitting from the online format of the module and accommodating various student needs and preferences, the course has no set start or end date. We recommend that students complete one theme per week, which includes listening to a podcast episode (around 30 min) and engaging with the associated learning activities which in total requires approx. 45 min per theme. Students will be advised to complete the full online module in 4 months.

## 3 Discussion

### 3.1 Strengths and limitations

The design of the complex ABC-uni intervention has several strengths.

The development of a robust program theory and comprehensive conceptual framework, incorporating recommendations from health promoting charters and the socio-ecological model of MHP, provides an exhaustive understanding of mental well-being and a whole university approach to MHP. Consistency in critical actions for health promotion adds to the coherence of the multi-level and multi-component intervention and enables easier replication and adaptation across diverse contexts as suggested by the MRC guidelines for complex interventions. The utilization of the ABC-framework offers both theoretical and research-based guidance, providing a structured approach to intervention components development and local adaptation. Furthermore, participants are recognized as change and health agents, with a focus on capacity building, co-creation, and action learning, hopefully fostering empowerment and sustainability. Finally, the intervention is not only focused on enhancing individual well-being but also contributes to a collective culture of well-being.

There are also several limitations. A fundamental challenge is the time-consuming nature of developing and implementing multi-level interventions. While such investments are recommended and worthwhile, attracting funding for long-term interventions can also be difficult. More specific limitations are included in the following SWOT analysis of the ABC-uni intervention in this first stage of the process. A SWOT analysis provides a structured methodology for evaluating internal strengths and weaknesses, alongside external opportunities and threats (77). This approach is fundamental in informing strategic decision-making processes and ensuring the best options for future implementation and efficacy of the intervention at the university. During the SWOT analysis, we adhered to the MRC framework recommendations, which advocate for the consideration of six core components: context, program theory, stakeholders, uncertainty, intervention refinement, and economic considerations (48). At this particular phase of the intervention, our focus primarily centered on the first three core components.

In evaluating the potential success, several, critical factors are considered at all levels of the program theory and the context of the ABC-uni: leadership support, resources to develop and deliver intervention components, and student and staff engagement and involvement. Identified weaknesses involve recognizing limited awareness of the intervention components among students, staff and faculty members, difficulties in engaging participants, resource constraints for creating awareness of the intervention, other compulsory commitments or obligations that hinders participation, lack of readiness for implementation within the organization, and challenges with evaluation intervention components, which may impede the implementation.

Regarding opportunities, there is a growing interest within the university context and among stakeholders to prioritize mental health promotion and integrate it as a key focus in higher education. This is expressed through leadership support, in strategic documents from the university sector and interest from students and staff. Several threats are at stake too. Ongoing reforms in the university sector may impact resource allocation and prioritize other initiatives over MHP. We also anticipate resistance from stakeholders who may be skeptical or resistant to adopting new approaches to MHP, as it is relatively new within the university setting. Finally, external factors such as the economic downturn and societal uncertainty due to wars, climate crisis etc. may also impact uncertainty and the economic situation, potentially affecting the engagement and support to initiatives like this.

### 3.2 Implications for policy, practice and research

The suggested conceptual framework for MHP at the university and its further development and concretization in the logic model holds implications for policy, research, and practice in the realm of MHP within higher education. Regarding implications for policy and practice, this study can contribute to shaping and advancing university well-being policies and actions as it contributes to the existing gap by providing a comprehensive mental well-being promotion strategy at the university context. The focus of our study aligns with the evolving landscape of evidence-based practices and contributes to the continuous improvement of interventions aimed at promoting mental well-being with a special focus on the university setting. Beyond the university setting, the findings can have broader implications for the wider community and society. By nurturing future generations with enhanced life skills and mental well-being literacy, universities play a pivotal role in shaping individuals who can positively contribute to various aspects of society. Regarding implications for research, the suggested conceptual framework could be used to inspire other research projects focusing on mental health and wellbeing in higher education settings. In this study, the primary emphasis is on enhancing mental well-being literacy among students, and subsequent research could explore avenues for promoting overall well-being among the university workforce.

The next important step will be to conduct phase two in the complex intervention framework focusing on feasibility and piloting of the ABC-uni. During this phase, the intervention will be tested in a feasibility study to measure its main uncertainties and assess its practicality, acceptability, and potential effectiveness. Data gathered during Phase II inform adjustments to the intervention before proceeding to larger-scale implementation and evaluation study. A future feasibility study of the ABC-uni intervention will create insight into the components’ practicality, adaptability, and perceived impact. Nevertheless, the complexity of the intervention will entail several challenges along the evaluation plan and therefore, ensuring the robustness and reliability along the evaluation design of the program theory and each component will be critical.

## 4 Ethics

Delivery of the intervention components will adhere to ethical guidelines and principles for conducting research involving human subjects to ensure the rights, well-being, and privacy of participants, as well as the integrity and credibility of the research. Thus, the study will follow the 1975 Helsinki Declaration as revised in 2008, APA ethical standards in publishing as well as national ethical guidelines. This study has received ethical approval for the components of both the online and elective courses from the Ethics Committee at the Department of Psychology, University of Copenhagen (approval reference number IP-EC-07022023 for the online module and approval reference number IP-EC-23062023-01 for the elective course).

Participation in the various components of ABC-uni intervention is anticipated to contribute to an increased awareness of mental health and well-being among participants. Throughout all study components, participants will be explicitly informed that the focus is on resources designed to foster mental well-being, as opposed to addressing the treatment of poor mental health or mental illness. Information regarding available support services, such as on-campus student counseling, will be provided to participants to ensure they are aware of the resources at their disposal.

Participants will be provided with comprehensive information, including the purpose, procedures, potential risks, and benefits of the study, through an information sheet and a consent form. Their participation in the evaluation is contingent upon providing explicit consent. This ensures that participants are well-informed and voluntarily agree to be a part of the study. Participation in the study components and the subsequent evaluation is completely voluntary.

## References

[1] Baik C Larcombe W Brooker A. How universities can enhance student mental wellbeing: the student perspective. High Educ Res Dev. (2019) 38:674–87. doi: 10.1080/07294360.2019.1576596

[2] Hammoudi Halat D Soltani A Dalli R Alsarraj L Malki A. Understanding and fostering mental Health and well-being among university faculty: a narrative review. J Clin Med. (2023) 12:4425. doi: 10.3390/jcm12134425, PMID: 37445459 PMC10342374

[3] Jürgensen I-N Koch P Nock AM Petersen-Ewert C. Health of (dual) health professional students in German-speaking countries: a scoping review. Front Public Health. (2023) 11:1243324. doi: 10.3389/fpubh.2023.1243324, PMID: 37794890 PMC10546053

[4] WHO . World mental health report: Transforming mental health for all. Geneva: World Health Organization (2022).

[5] Koushede V Donovan R. The application of salutogenesis to communitywide mental health promotion: the act-belong-commit/ABC’s of mental Health campaign and framework In: Mittelmark MB , editor. Handbook of Salutogenesis. Cham: Springer (2022)

[6] Antonovsky A . Unraveling the mystery of health: How people manage stress and stay well. San Francisco: Jossey-Bass (1987). 218 p.

[7] WHO . Health promotion glossary of terms. Geneva: World Health Organization (2021).

[8] Barry MM . Concepts and principles of mental health promotion In: Barry MM Clarke AM Petersen I Jenkins R, editors. Implementing mental Health promotion. Cham: Springer Nature (2019). 3–34.

[9] Bronfenbrenner U . The ecology of human development. London: Harvard University Press (1979). 352 p.

[10] Michaels C Blake L Lynn A Greylord T Benning S. Mental health and well-being ecological model. Twin Cities: Center for Leadership Education in Maternal & Child Public Health, University of Minesota (2024).

[11] Bücker S Nuraydin S Simonsmeier BA Schneider M Luhmann M. Subjective well-being and academic achievement: a meta-analysis. J Res Pers. (2018) 74:83–94. doi: 10.1016/j.jrp.2018.02.007

[12] Conley CS Durlak JA Kirsch AC. A meta-analysis of universal mental health prevention programs for higher education students. Prev Sci. (2015) 16:487–507. doi: 10.1007/s11121-015-0543-1, PMID: 25744536

[13] Riva E Freeman R Schrock L Jelicic V Ozer C-T Caleb R. Student wellbeing in the teaching and learning environment: a study exploring student and staff perspectives. High Educ Stud. (2020) 10:103. doi: 10.5539/hes.v10n4p103

[14] International Union for Health Promotion and Education . Critical actions for mental health promotion. Paris: International Union for Health Promotion and Education (2021).

[15] Fernandez A Howse E Rubio-Valera M Thorncraft K Noone J Luu X . Setting-based interventions to promote mental health at the university: a systematic review. Int J Public Health. (2016) 61:797–807. doi: 10.1007/s00038-016-0846-4, PMID: 27364779

[16] Dooris M Cawood J Doherty S Powell S. Concept, model and framework for applying the healthy settings approach within higher education in England Healthy Universities (2010). Available at: https://clok.uclan.ac.uk/5632/1/5632_HU-Final_Report-FINAL_v2.pdf

[17] Lindsay BL Bernier E Boman J Boyce MA. Understanding the connection between student wellbeing and teaching and learning at a Canadian research university: a qualitative student perspective. Pedagogy Health Promot. (2023) 9:5–16. doi: 10.1177/23733799221089578

[18] Geertshuis SA . Slaves to our emotions: examining the predictive relationship between emotional well-being and academic outcomes. Active Learn High Educ. (2019) 20:153–66. doi: 10.1177/1469787418808932

[19] Turner M Holdsworth S Scott-Young CM. Resilience at university: the development and testing of a new measure. High Educ Res Dev. (2016) 36:386–400. doi: 10.1080/07294360.2016.1185398

[20] Keeling RP . An ethic of care in higher education: well-being and learning. J Coll Character. (2014) 15:141–8. doi: 10.1515/jcc-2014-0018

[21] Hughes G Spanner L. The university mental Health Charter. Leeds: Student Minds (2019).

[22] Okanagan Charter . An international Charter for Health promoting Universities and colleges. (Vancouver: University of British Columbia Library) (2015). Available at: https://open.library.ubc.ca/cIRcle/collections/53926/items/1.0132754

[23] WHO . Ottawa charter for health promotion. Ottawa: World Health Organization (1986).

[24] Universities UK . Stepchange. Woburn House, London: Mentally healthy Universities (2020). Available at:https://www.universitiesuk.ac.uk/sites/default/files/field/downloads/2021-07/uuk-stepchange-mhu.pdf

[25] Cefai C Simões C Caravita S. A systemic, whole-school approach to mental health and well-being in schools in the EU. NESET report. Luxembourg: Publications Office of the European Union (2021).

[26] Hughes G Panjwani M Tulcidas P Byrom N. Student mental health: the role and experiences of academics. Student Minds. (2018). 1–60. Available at: https://www.studentminds.org.uk/uploads/3/7/8/4/3784584/180129_student_mental_health__the_role_and_experience_of_academics__student_minds_pdf.pdf

[27] Houghton A-M Anderson J. Embedding mental wellbeing in the curriculum: Maximising success in higher education. York: Higher Education Academy (2017). 44 p.

[28] Oades LG . Wellbeing literacy: the missing link in positive education In: White M Slemp GR Murray AS, editors. Future directions in well-being. Cham: Springer (2017). 169–73.

[29] Upsher R Nobili A Hughes G Byrom N. A systematic review of interventions embedded in curriculum to improve university student wellbeing. Educ Res Rev. (2022) 37:100464. doi: 10.1016/j.edurev.2022.100464

[30] Piper R Emmanuel T. Co-producing mental health strategies with students: A guide for the higher education sector. Leeds: Student Minds (2019).

[31] Lister K Riva E Kukulska-Hulme A Fox C. Participatory digital approaches to embedding student wellbeing in higher education. Front Educ. (2022) 7:7. doi: 10.3389/feduc.2022.924868

[32] Zandvliet DB Stanton A Dhaliwal R. Design and validation of a tool to measure associations between the learning environment and student well-being: the healthy environments and learning practices survey (HELPS). Innov High Educ. (2019) 44:283–97. doi: 10.1007/s10755-019-9462-6

[33] Lister K Andrews K Buxton J Douce C Seale J. Assessment, life circumstances, curriculum and skills: barriers and enablers to student mental wellbeing in distance learning. Front Psychol. (2023) 14:14. doi: 10.3389/fpsyg.2023.1076985, PMID: 36814661 PMC9940708

[34] Koushede V Nielsen L Meilstrup C Donovan R. From rhetoric to action: adapting the act-belong-commit mental health promotion programme to a Danish context. Int J Ment Health Promot. (2015) 17:22–33. doi: 10.1080/14623730.2014.995449

[35] Hinrichsen C Koushede VJ Madsen KR Nielsen L Ahlmark NG Santini ZI . Implementing mental health promotion initiatives: process evaluation of the ABCs of mental Health in Denmark. Int J Environ Res Public Health. (2020) 17:5819. doi: 10.3390/ijerph17165819, PMID: 32796754 PMC7460269

[36] Santini ZI Nelausen MK Kusier AO Hinrichsen C Schou-Juul F Madsen KR . Impact evaluation of the “ABCs of mental Health” in Denmark and the role of mental health-promoting beliefs and actions. Ment Health Soc Incl. (2022) 26:271–91. doi: 10.1108/MHSI-03-2022-0014

[37] Antonovsky A . The salutogenic model as a theory to guide health promotion. Health Promot Int. (1996) 11:11–8. doi: 10.1093/heapro/11.1.11

[38] Bukdahl D . Good solutions to difficult social problems (in Danish: Gode løsninger på svære sociale problemer). Copenhagen: Samfundslitteratur (2021). 300 p.

[39] Meilstrup C Santini Z Koushede V Nelausen MK Hinrichsen C Schou-Juul F . Developing a principles-based approach to mental health promotion informed by a decade of practice, evaluation and research within the ABCs of mental health in Denmark. [In preparation.]

[40] Patton MQ . Principles-focused evaluation - the GUIDE. New York: Guilford Press (2018). 435 p.

[41] OECD . How to make societies thrive? Coordinating approaches to promote well-being and mental health. Paris: OECD Publishing (2023).

[42] Barry M . Capacity building for the future of health promotion. Promot Educ. (2008) 15:56–8. doi: 10.1177/1025382308097700 19066241

[43] NSW Health Department . A framework for building capacity to improve health. Sydney: NSW Health Department (2001). 35 p.

[44] Nutbeam D Muscat DM. Health promotion glossary 2021. Health Promot Int. (2021) 36:1578–98. doi: 10.1093/heapro/daaa157, PMID: 33822939

[45] Hernantes N Bermejo-Martins E Øvergård KI Pumar-Mendez MJ Lopez-Dicastillo O Iriarte-Roteta A . Theory-based capacity building intervention for intersectoral action for health at local governments: an exploratory pilot study. J Adv Nurs. (2022) 78:1798–814. doi: 10.1111/jan.15247, PMID: 35436006 PMC9322672

[46] Guglielmin M Muntaner C O'Campo P Shankardass K. A scoping review of the implementation of health in all policies at the local level. Health Policy. (2018) 122:284–92. doi: 10.1016/j.healthpol.2017.12.005 29305241

[47] O'Cathain A Croot L Duncan E Rousseau N Sworn K Turner KM . Guidance on how to develop complex interventions to improve health and healthcare. BMJ Open. (2019) 9:e029954. doi: 10.1136/bmjopen-2019-029954, PMID: 31420394 PMC6701588

[48] Skivington K Matthews L Simpson SA Craig P Baird J Blazeby JM . A new framework for developing and evaluating complex interventions: update of Medical Research Council guidance. BMJ. (2021) 374:n2061. doi: 10.1136/bmj.n2061, PMID: 34593508 PMC8482308

[49] Faculty of Social Sciences, Univeristy of Copenhagen . About the Faculty of Social Sciences. Faculty of Social Sciences, University of Copenhagen (2024) Available at:https://socialsciences.ku.dk/faculty/ Accessed April 18, 2024

[50] Danish Agency for Higher Education and Science. Study in Denmark (2024) Available at:https://studyindenmark.dk/ [Accessed April 18, 2024]

[51] Barry MM . Generic principles of effective mental health promotion. Int J Ment Health Promot. (2007) 9:4–16. doi: 10.1080/14623730.2007.9721834

[52] Kaluzny AD . Change in health care settings In: Trohanis P , editor. Ideas in change. Chapel Hill: Frank Porter Graham Child Development Center, University of North Carolina (1981). 43–65.

[53] Frechtling JA . Logic modeling methods in program evaluation John Wiley & Sons (2007).

[54] Pychyl TA Flett GL Long M Carreiro E Azil R. Faculty perceptions of mattering in teaching and learning: a qualitative examination of the views, values, and teaching practices of award-winning professors. J Psychoeduc Assess. (2022) 40:142–58. doi: 10.1177/07342829211057648

[55] Flett G Khan A Su C. Mattering and psychological well-being in college and university students: review and recommendations for campus-based initiatives. Int J Ment Health Addict. (2019) 17:667–80. doi: 10.1007/s11469-019-00073-6

[56] Department of Psychology, Univerisity of Copenhagen . Together at Psychology [In Danish: Sammen på PSYK] (2024) Available at:https://psy.ku.dk/abc/sammen-paa-samf/sammen-paa-psyk/ Accessed April 18, 2024.

[57] Oades LG Jarden A Hou H Ozturk C Williams P G RS . Wellbeing literacy: a capability model for wellbeing science and practice. Int J Environ Res Public Health. (2021) 18:719. doi: 10.3390/ijerph18020719, PMID: 33467630 PMC7829945

[58] University of Copenhagen . Day journey movie. (2024). Available at: https://psy.ku.dk/abc/sammen-paa-samf/dagsrejsen/ (Accessed April 18, 2024).

[59] Wang C Burris MA. Photovoice: concept, methodology, and use for participatory needs assessment. Health Educ Behav. (1997) 24:369–87. doi: 10.1177/109019819702400309, PMID: 9158980

[60] Goodhart FW Hsu J Baek JH Coleman AL Maresca FM Miller MB. A view through a different lens: photovoice as a tool for student advocacy. J Am Coll Heal. (2006) 55:53–6. doi: 10.3200/jach.55.1.53-56, PMID: 16889316

[61] Cornell J Kessi S Ratele K. Examining the dynamics of belonging and alienation in higher education through photovoice. Health Promot Pract. (2022) 23:325–30. doi: 10.1177/15248399211054779, PMID: 35285328

[62] Danker J Strnadová I Cumming TM. Picture my well-being: listening to the voices of students with autism spectrum disorder. Res Dev Disabil. (2019) 89:130–40. doi: 10.1016/j.ridd.2019.04.005, PMID: 30986668

[63] Hall WJ Witkemper KD Rodgers GK Waters EM Smith MR. Activating adult allies from a rural community on lesbian, gay, bisexual, transgender, and queer student issues in school using photovoice. J Gay Lesbian Soc Serv. (2018) 30:49–63. doi: 10.1080/10538720.2017.1408517, PMID: 30828237 PMC6395050

[64] Harper D . Talking about pictures: a case for photo elicitation. Vis Stud. (2002) 17:13–26. doi: 10.1080/14725860220137345

[65] Phillips L Christensen-Strynø MB Frølunde L. Arts-based co-production in participatory research: harnessing creativity in the tension between process and product. Evid Policy. (2022) 18:391–411. doi: 10.1332/174426421x16445103995426

[66] Madsen CS Nelausen MK Bermejo-Martins E Meilstrup CB Pollard C Nielsen L. Qualitative photovoice study: students’ perspectives on mental health promoting resources at a university campus. Under review with BMC Public Health, 2024.

[67] Cage E Jones E Ryan G Hughes G Spanner L. Student mental health and transitions into, through and out of university: student and staff perspectives. J Furth High Edu. (2021) 45:1076–89. doi: 10.1080/0309877X.2021.1875203

[68] Hill M Farrelly N Clarke C Cannon M. Student mental health and well-being: overview and future directions. Ir J Psychol Med. (2020) 11:1–8. doi: 10.1017/ipm.2020.110, PMID: 33243317

[69] Queroue M Pouymayou A Pereira E Tzourio C González-Caballero JL Montagni I. An interactive video increasing French students' mental health literacy: a mixed-methods randomized controlled pilot study. Health Promot Int. (2023) 38:daab202. doi: 10.1093/heapro/daab202, PMID: 34897453

[70] Riva E Stewart-Brown S Rahman Y Gerson J Ashworth S. Can an academic, interdisciplinary intervention help to solve wellbeing issues among higher education students? Interdisciplinary learning and teaching: practice and pedagogies. Ethics Press: Cambridge. Forthcoming. https://ethicspress.com/collections/forthcoming-titles/products/interdisciplinary-learning-and-teaching

[71] Griebler U Rojatz D Simovska V Forster R. Effects of student participation in school health promotion: a systematic review. Health Promot Int. (2014) 32:195–206. doi: 10.1093/heapro/dat090 24395957

[72] Davies EB Morriss R Glazebrook C. Computer-delivered and web-based interventions to improve depression, anxiety, and psychological well-being of university students: a systematic review and meta-analysis. J Med Internet Res. (2014) 16:e130. doi: 10.2196/jmir.3142, PMID: 24836465 PMC4051748

[73] Goozee R Barrable A Lubenko J Papadatou-Pastou M Haddad M McKeown E . Investigating the feasibility of MePlusMe, an online intervention to support mental health, well-being, and study skills in higher education students. J Ment Health. (2022) 12:1–11. doi: 10.1080/09638237.2022.2069699, PMID: 35549804

[74] Conley CS Durlak JA Shapiro JB Kirsch AC Zahniser E. A meta-analysis of the impact of universal and indicated preventive technology-delivered interventions for higher education students. Prev Sci. (2016) 17:659–78. doi: 10.1007/s11121-016-0662-3, PMID: 27225631

[75] Hood B Jelbert S Santos LR. Benefits of a psychoeducational happiness course on university student mental well-being both before and during a COVID-19 lockdown. Health Psychol Open. (2021) 8:2055102921999291. doi: 10.1177/2055102921999291, PMID: 33796324 PMC7983240

[76] Yaden DB Claydon J Bathgate M Platt B Santos LR. Teaching well-being at scale: an intervention study. PLoS One. (2021) 16:e0249193. doi: 10.1371/journal.pone.0249193, PMID: 33852597 PMC8046248

[77] Ahmadi Q Danesh H Makharashvili V Mishkin K Mupfukura L Teed H . SWOT analysis of program design and implementation: a case study on the reduction of maternal mortality in Afghanistan. Int J Health Plann Manag. (2016) 31:247–59. doi: 10.1002/hpm.2288, PMID: 25950757

